# High Carbohydrate 19-9 Antigen Serum Levels in Patients with Nonmelanoma Skin Cancer and Primary Occult Cancer

**DOI:** 10.3390/biomedicines8080265

**Published:** 2020-08-03

**Authors:** Giulia Malaguarnera, Saverio Latteri, Roberto Madeddu, Vito Emanuele Catania, Gaetano Bertino, Rosario Emanuele Perrotta, Francesco Dinotta, Michele Malaguarnera

**Affiliations:** 1 Department of Biomedical and Biotechnological Science, University of Catania; 95123 Catania, Italy; giulia.malaguarnera@live.it (G.M.); michele.malaguarnera@gmail.com (M.M.); 2 Department of Medical, Surgical Sciences and Advanced Technologies “G.F. Ingrassia”, University of Catania, 95123 Catania, Italy; savlat@tiscali.it; 3 Department of Biomedical Sciences, University of Sassari, 07100 Sassari, Italy; rmadeddu@uniss.it; 4 Department of Experimental and Clinical Medicine, University of Catania, 95123 Catania, Italy; gaetanobertinounict@gmail.com; 5 Department of General Surgery and Medical-Surgery Specialties, University of Catania, 95100 Catania, Italy; r.perrotta@unict.it (R.E.P.); f.dinotta@unict.it (F.D.)

**Keywords:** carbohydrate 19-9 antigen, nonmelanoma skin cancer, squamous cell carcinoma, basal cell carcinoma, cancer, multiple tumours, occult primary malignancies

## Abstract

Background: Non-melanoma skin cancers (NMSC), despite having a favourable prognosis, present an increased risk of occult malignancies. The aim of this study was the evaluation of the usefulness of the mucinous marker carbohydrate 19-9 antigen (CA 19-9) in the diagnosis of occult cancers. (1) Patients and Methods: This is a case control study in which 480 patients with NMSC and 480 matched control subjects with dermatitis were enrolled; 208 patients with NMSC showed upper-normal CA 19-9 values, and 272 showed under-normal CA 19-9 values. (2) Results: The 208 patients positive for CA 19-9 included 87 with basal cell carcinoma (BCC) and 121 with squamous cell carcinoma (SCC). The 272 patients negative for CA 19-9 included 107 with BCC and 165 with SCC. For the SCC patients, CA 19-9 serum levels were significant in 121 of the patients (positive), 66 of which were affected by cancer; CA 19-9 was within the normal range in 165 patients, of which 30 were diagnosed with cancer. In the SCC patients, the CA 19-9 sensitivity was 68%, the specificity was 70%, the positive predictive value (PPV) was 54% (95%) and the negative predictive value (NPV) was 81%. In the BCC patients, the CA 19-9 sensitivity was 70%, the specificity was 66%, the PPV was 48% and the NPV was 83%. In the dermatitis patients (controls), we observed 121 patients that were CA 19-9 positive, with 15 malignancies, and 359 CA 19-9-negative patients, with three malignancies. (3) Conclusions: To confirm the association between CA 19-9 and an elevated risk of malignancies in NMSC, prospective cohort studies should be performed.

## 1. Introduction

Non-melanoma skin cancers (NMSCs) are the most common malignancy worldwide with more cases diagnosed annually in United States than of prostate, colorectal, ovarian, lung and breast cancers combined. Hence, they are a growing public health problem due to their increasing incidence and attendant medical care costs [1,2,3].

NMSC, which consists of two histological subtypes of basal cell carcinoma (BCC) and squamous cell carcinoma (SCC), has been rising over the past decades. The number of people with NMSC increased from 2.4 million to 3.3 million from 2006 to 2012 in the USA [4,5].

NMSCs represent a significant economic burden to health services and can cause significant morbidity [6]. Despite such a favourable prognosis, there is evidence that NMSC may be a marker of increased risks of other adverse health outcomes and other malignancies [7,8,9].

Several studies have reported that NMSC patients have higher subsequent or prior diagnoses of second primary malignancies by about 20–60% [10,11,12].

Tumour markers are widely used in oncology and have become an integral part of the clinical management of many different malignancies, and they are used for screening, diagnosing, staging and prognosis. Serum tumour markers are non-invasive, low-cost, popular and convenient tools for widespread clinical application. Among various tumour markers, carbohydrate antigen 19-9 (CA 19-9) is a tumour-associated, not a tumour-specific, antigen [13].

CA 19-9 is also called sialyl Lewis antigen (Le-a) and in the population of individuals that receive regular health examinations can be observed to be elevated in healthy individuals. Healthy subjects with a Le-a positive secretor status may show physiologically elevated CA 19-9 concentrations in all types of secretions (e.g., saliva, bronchial sputum, gastric secretions and bile juice) in contrast with patients with a Le-a negative status, who have little or no CA 19-9 in their serum [14,15].

Obesity, diabetes, alcohol consumption and smoking in Le-a positive individuals may increase the serum level of CA 19-9 [16].

CA19-9 is a carbohydrate antigen, expressed in tissues as a monosialoganglioside. It is a mucous protein defined from the culture medium, identified by a hybridoma prepared from murine spleen cells immunized with a human colorectal cancer cell line [17].

CA 19-9 is synthesized by normal human pancreatic and biliary ductular cells, as well as by gastric, colonic, endometrial and salivary epithelia. This high-molecular-weight glycoprotein is expressed by several epithelial cancers as well as in normal pancreatic and biliary ductal epithelial cells, and it is also detectable in salivary mucus [18].

CA 19-9 is considered the best serum marker for pancreatic cancer because of its sensitivity of 80% and specificity of 90%. High CA 19-9 levels have been associated with unresectable lesions and poor prognosis in patients with pancreatic carcinoma [19,20,21,22].

CA19-9 levels also increase in non-neoplastic and organ-specific diseases [23] such as achalasia, acute hepatitis, hepatic cirrhosis and respiratory diseases and in systemic diseases such as diabetes mellitus, rheumatic and autoimmune disorders, simple liver cysts, severe steatosis, autoimmune hepatitis and chronic alcoholic hepatitis [24,25,26,27,28,29,30,31,32].

Elevation above the upper normal limit (URL 39 IU/mL) of CA 19-9 in serum is frequently found, but when a marked elevation is present concern for a possible occult primary malignancy is raised. For this reason, we analysed serum CA 19-9 levels and imaging techniques that can provide important screening and predictive functions in the care of NMSC patients. The aim of this study was the evaluation of the usefulness of the mucinous marker CA 19-9 in the diagnosis of NMSC and of occult cancers in this condition.

## 2. Methods and Patients

### 2.1. Patients

In this case control study, 480 patients with NMSC and 480 control subjects were included, regardless of sex, age and geographic location. Patients and control subjects underwent abdominal ultrasound scans and, when needed, computerized tomography. Only standard diagnostic and therapeutic procedures were employed. Suspicion of skin tumours was determined by clinical examination, and the diagnosis was confirmed by the histopathology of the excised specimens.

We collected relevant information on demographic factors, personal histories of any cancer including skin cancer, family histories of skin phototypes, and professional and recreational exposure to sunlight and/or UV using a questionnaire. Eligible patients for this study were those who were 40 years of age or older, with the presence of nonmelanoma skin cancer. The matching criteria were the country, sex, and date of blood collection (± one year relaxed to ± five years) for patients that were admitted to Cannizzaro Hospital. Clinical evaluations and haematochemical, virological, instrumental and histological analysis were performed on these patients. Ineligible patients were those with a prior history of another cancer, severe jaundice, pulmonary renal chronic diseases, prostatic diseases, autoimmune diseases, immunosuppressed states, human papilloma virus and infection.

Demographic information, including patients’ sex and date of birth, was obtained at the time of enrolment. Data on smoking status estimates (ever/never), and height and weight for body mass index (BMI) calculation were also obtained (Table 1).

### 2.2. Methods

Fasting blood was collected by a puncture of the cubital vein in the morning and processed by centrifugation. NMSC was subdivided into BCC and SCC.

The reference population of this study was situated in relatively homogeneous areas regarding sun exposure. The average number of sunny hours per year is between 1200 to 1800 h. The patients and control dermatitis subjects were undergoing abdominal echography and, if needed, computed tomography.

Every subject in this study underwent clinical analysis and CA 19-9 analysis.

For each case of nonmelanoma skin cancer, one CA 19-9 control was randomly chosen from the risk sets consisting of all the cohort members alive and free of nonmelanoma.

### 2.3. Tumour Marker Assay

Blood samples (10 mL each) were taken from each patient then were processed and stored at −80 °C until examination. Serum assays for CA 19-9 were performed with Immulite 2000 GI-MA. Immulite 2000 GI-MA [Siemens Medical Solutions-Diagnostics-USA (ex DPC Instrument Systems Division—New Jersey (USA)], is a solid phase, two-site sequential chemiluminescent immunometric assay [33].

### 2.4. Laboratory Analysis

The following serum analyses were performed: urea, creatinine, aspartate aminotransferase and alanine aminotransferase (AST and ALT), gamma-glutamyl transferase (gGT), alkaline phosphatase, prothrombin, fibrinogen, and prothrombin time (PT).

The distinction between benign and malignant disease was based on the clinical course, instrumental examination, and pathology. The instrumental examination included ultrasonography, computerized tomography scans and magnetic resonance imaging. Pathology samples were obtained, when possible, from percutaneous biopsy or fine-needle aspiration cytology, endoscopic biopsy/brushing or surgical specimens.

### 2.5. Ethics

All sensitive data were collected and protected in respect of present privacy statements. The patients enrolled in this study were admitted to Cannizzaro Hospital during the period from 2009 to 2016. All patients and control subjects gave their informed written consent for study participation and for each invasive procedure. The study was conducted in line with the ethical principles set out in the Helsinki Declaration. The study protocol was approved by the ethics Committee of Cannizzaro Hospital.

## 3. Statistical Analysis

Student’s *t*-test and the Chi-square test were used to compare continuous and categorical data. Numerical variables were expressed as median and range. The Kolmogorov–Smirnov test was used to assess the normality of the distribution of the CA 19-9 serum levels. A comparison between serum levels was performed by means of the Mann–Whitney U test. Differences in the frequencies for categorical variables were assessed using Fisher’s exact test. We consider a two-sided *p* value of <0.05 significant.

We analysed the data obtained and calculated the sensitivity, specificity, positive predictive value (PPV), negative predictive value (NPV), positive likelihood ratio (PLR) and negative likelihood ratio (NLR).

All data management and statistical calculations were performed using the SPSS 15.0 statistical package (Chicago, IL, USA).

## 4. Results

### 4.1. Basal Characteristics

The basal characteristics of the patients with NMSC are described in Table 1 and Table 2.

The ages of the 480 patients ranged from 65 to 80 years. Among these 480 subjects, NMSC was located on the faces of 240 patients, on the chests of 120 patients, on legs in 46 patients, on the scalps in 39 patients, on hands in 14 patients, on buttocks in 13 patients and on the neck in 8 patients. A total of 208 were positive for CA 19-9, and 272 were negative. Regarding the histology, 286 patients were affected by SCC, and 194, by BCC. Among the 208 patients positive for CA 19-9, 87 had BCC and 121 had SCC. Of the 272 patients negative for CA 19-9, 107 had BCC and 165 had SCC.

### 4.2. CA 19-9 in NMSC

*CA 19-9 in the Basal Cell Carcinoma Patients*

The CA 19-9 serum levels in the BCC patients were significant in 87 subjects (positive), and 42 of the CA 19-9-positive patients had another cancer; CA 19-9 was absent within the normal range in 107 subjects, 18 of which had cancer.

*CA 19-9 in SCC Patients*

In the SCC patients, the CA 19-9 serum levels were significant in 121 patients (positive), and 66 of the patients were affected by cancer; CA 19-9 was absent in 165 patients, 30 of which had cancer.

### 4.3. CA 19-9′s Predictive Value in NMSC

For BCC, CA 19-9′s sensitivity is 70% (95% CI: 58–81%), its specificity is 66% (95% CI: 58–74%), its PPV is 48% (95% CI: 43–54%) and its NPV is 83% (95% CI: 80–87%). For SCC, CA 19-9′s sensitivity is 68% (95% CI: 59–78%), its specificity is 70% (95% CI: 64–77%), its PPV is 54% (95% CI: 50–60%) and its NPV is 81% (95% CI: 79–82%). In the dermatitis patients (controls), we observed that 15 of the 121 CA 19-9-positive subjects displayed malignancies and 3 of the 359 CA 19-9-negative patients showed malignancies (Table 3).

### 4.4. Comparison with Groups

(A) SCC vs. BCC

In SCC compared to BCC patients, we observed higher serum levels of AST, ALT, gamma-GT, ALP and serum creatinine (*p* < 0.001).

(B) SCC vs. Dermatitis

In SCC compared to dermatitis patients, we observed higher serum levels of AST (*p* < 0.001), ALT (*p* < 0.001), gamma-GT (*p* < 0.001), ALP (*p* < 0.005), PT (*p* < 0.005) and urea (*p* < 0.001).

(C) CC vs. Dermatitis

In BCC compared to dermatitis patients, we observed higher serum levels of ALT (*p* < 0.001), gamma-GT (*p* < 0.001), ALP (*p* < 0.005), glucose (*p* < 0.001), urea (*p* < 0.001) and CA 19-9 (*p* < 0.001).

### 4.5. Association of BCC, SCC and Dermatitis with Occult Malignancies

(a) In the BCC group, we observed that 23.3% had occult malignancies of the pancreas; 18.3%, malignancies of the prostate; 16.6%, both breast and lung malignancies; 6.6%, malignancies of the bladder and uterus; 3.3%, neoplasms of the colon and rectum; and 1.7%, neoplasms of the gallbladder, stomach and oesophagus.
(b) In SCC group, we observed that 18.7% had malignancies of the prostate; 16.6%, malignancies of the pancreas; 14.5%, malignancies of the breast and lung; 10.4%, malignancies of the bladder; 6.2%, neoplasms of the uterus; 4.17%, malignancies of the stomach, colon and rectum; 3.13%, neoplasms of the gallbladder; 2.08%, hepatocellular carcinoma; and 1.04%, neoplasms of the oesophagus.
(c) In the dermatitis group, we observed that 27% had breast cancer; 16.6%, pancreas cancer; 11.11%, bladder, uterus and lung cancer; and 5.56%, prostate, gallbladder, stomach and colon cancer (Table 3).

In our study, we found 60 occult malignancies in BCC; in 42 (70%), CA 19-9 was >39 U/L, and in 18 (30%), it was below 39 U/L (*p* < 0.01). In the SCC patients, we found 96 occult malignancies; in 66 (68.75%), CA 19-9 was >39 U/L, and in 30 (31.25%), it was below 39 U/L (*p* < 0.01).

In dermatitis patients, we found 18 occult malignancies; in 15 (83.33%), CA 19-9 was >39 U/L, and in 3 (16.67%), it was below 39 U/L (*p* < 0.01). These data are summarized in Table 4.

## 5. Discussion

Elevated CA 19-9 may be found in patients with benign as well as malignant disease.

Multiple studies have shown that patients with elevated levels of CA 19-9 have a worse prognosis than those with low levels [34].

CA19-9 is a mucin-type glycoprotein present only in trace amounts in the serum and normally absent in other tissues such as the pulmonary epithelium, perialveolar interstitial space and liver. The results of the population-based study suggest that there may be an increased baseline risk of BCC in certain individuals with Intestinal Bowel Disease (IBD) such as men with Crohn Disease (CD).

CA 19-9 is expressed as a monosialoganglioside on secreted mucus glycoproteins in colorectal and pancreatic tumours. It can also be detected on mucin in patient sera [35].

Other sources include the normal pancreas, bile ducts, and gastric, colic, endometrial and salivary epithelia.

Elevation above the upper normal limit of CA 19-9 in serum is frequently found, raising concern for possible occult malignant disease. Inflammation contributes to the elevation of the CA 19-9 value, and it can be assessed by monitoring the acute-phase proteins; one of these is the C-reactive protein (CRP), which rises in response to infection, injury and neoplasms [36,37,38].

NMSC generally has a favourable prognosis, and rarely is it fatal, but the documented increased risk of other malignancies suggests the need to use the tumour markers.

Growing evidence raises the possibility that NMSC may be associated with increased risks of other malignancies and with increased mortality [39,40].

Two potential pathways that may relate NMSC risk to overall cancer risk are DNA repair and inflammatory and/or immune response pathways.

The CA 19-9 antigen circulates at low levels, normally about 39 U/mL among dermatitis subjects. Elevated levels have also been seen in benign inflammatory diseases of the biliary tract [41,42].

Previous studies showed that CA 19-9 levels have no value in screening for asymptomatic individuals because the positive value was low [43,44,45,46].

In dermatitis subjects, we observed a positive predictive value of 13% and a negative predictive value of 99%.

Studies have also shown that a mucin bearing this antigen was detected more often in the sera of patients with pancreatic cancer than for any other gastrointestinal carcinoma, including colorectal cancer.

It is also found in the bile duct, ovarian mucinous cystadenocarcinomas and uterine adenocarcinomas. In our study, the CA 19-9 concentration at the cut-off point shows a sensitivity of 68% and specificity of 70% in SCC and a sensitivity of 70% and specificity of 66% in BCC for the diagnosis of occult malignancies in NMSC [47,48,49,50,51].

The positive predictive value (PPV) and negative predictive value (NPV) for NMSC with occult malignancies were reported, respectively, to be 48% and 83% for BCC and 54% and 81% for SCC.

Due to the uniformly poor outcomes in NMSC with gastrointestinal malignancies, extensive research has been dedicated to identifying better serum biomarkers. Many benign diseases, as well as malignancies, should be considered as possible causes of elevated CA 19-9 levels in asymptomatic subjects.

Therefore, more attention should be paid to these causative diseases in dermatitis patients, and further work for determining the aetiology should be performed in cases of elevated CA 19-9 levels.

Although these marker panels improve the detection of NMSC, they may not be applicable for actual screening or pre-diagnostic assessment for early detection.

The clinical interpretation of CA 19-9 measurements requires an appreciation of the normal biology of this tumour marker. Despite showing early promise, this tumour marker has failed to gain an established role in clinical practice, partly due to uncertainty surrounding the predictive value of a positive test. It is now appreciated that this marker is not exclusively associated with malignant processes, and elevated circulating levels have been reported in a wide range of benign conditions including liver disease, cholangitis and pancreatitis. The diagnostic significance of an elevated CA 19-9 level must be evaluated cautiously, while giving consideration to an individual’s clinical situation. CA 19-9 serum levels can be used as a marker for the follow-up of chronic organ-specific inflammations.

This study shows that is not possible to reliably distinguish benign from malignant disease processes on the basis of this tumour marker. This is particularly relevant in the presence of jaundice, and such patients should not be assumed to harbour a malignancy.

Thus, we would caution against the use of a single measurement of this marker in the routine follow-up of patients. A rising level in the absence of further jaundice is suggestive of an underlying malignancy, although a falling value does not exclude the diagnosis. The extensive overlap in CA 19-9 values between benign and malignant cases shown indicates that confident discrimination can rarely be made on the basis of a single measurement.

Although the sample size was large enough to exclude bias, our study was limited by a single-centre design and we did not evaluate the immunolocalization for the CA 19-9 in the biopsies. The lack of rigorous control for potential confounding factors, such as smoking history and alcohol intake, is a limitation. Cigarette smoking is causally associated with major causes of immune dysregulation and occult cancer [52].

Additional investigation is needed to characterize these associations and elucidate potential underlying mechanisms.

## 6. Conclusions

Elevated CA19-9 levels are not pathognomonic of pancreatic cancer [53].

When clinicians encounter NMSC patients with elevated CA 19-9 levels, they must still determine the cause of the elevated CA 19-9 levels and ascertain whether there is a malignancy related to the elevated marker and, if not, ascertain what other conditions may have caused the elevation.

Even through CA 19-9 is known to be a tumour marker for pancreatic tumours when elevated, CA 19-9 may be associated with various kinds of extra-pancreatic malignancies such as bile duct cancer, gastrointestinal tract cancer, Hepatocellular Carcinoma (HCC) and genitourinary tract cancer. The results show that CA 19-9 may be useful for diagnosing occult malignancy in NMSC patients.

Prospective studies with larger screened NMSC proportions are warranted to ascertain the most appropriate cut-off value for CA 19-9 and the exact proportion of malignancies in a screening setting. It represents a useful means for determining the prognosis of NMSC, a raised level providing indirect individual evidence of primary occult malignancy and the need for proper and immediate therapeutic intervention to decrease the risk of mortality. High CA 19-9 concentrations may influence survival and should lead to aggressive follow-up examinations.

The extensive overlap of the CA 19-9 values from the benign and malignant cases indicates that confident discrimination cannot be made on the basis of a single measurement.

## Figures and Tables

**Table 1:** Baseline clinical characteristics of the study cohorts.

Characteristics	Dermatitis	SCC	BCC
	(n.480)	(n.286)	(n.194)
Sex (Male/Female)	261/219	165/121	98/96
	(44%/56%)	(63.45%/36.55%)	(52.84%/47.16%)
Age (years)	65–80	66–80	65–80
Race	Caucasian	Caucasian	Caucasian
Smokers	44.2%	45.8%	45.6%
Dyspepsia	33.8%	34.1%	34.3%
Hypertension	25.2%	24.7%	25.0%
Alcohol	36.2%	34.5%	36.1%

**Table 2:** Parameters and biochemical characteristics of the study cohort (values are expressed as the mean ± SD). A: Comparison of dermatitis and SCC; B: Comparison of dermatitis and BCC; C: Comparison of SCC and BCC. Statistical significance: * *p* < 0.05, ** *p* < 0.001, NS = non-significant.

Characteristics	Dermatitis	SCC	BCC
	(n: 480)	(n: 286)	(n: 194)
Body-mass index (kg/m^2^)	26.21 ± 2.70 * A/** B	26.80 ± 2.60 * C	27.60 ± 2.40
Systolic blood pressure (mmHg)	138.00 ± 8.20 NS A/NS B	138.00 ± 9.80 NS C	137.00 ± 7.10
Diastolic blood pressure (mmHg)	86.70 ± 8.10 NS A/NS B	88.00 ± 9.50 * C	86.00 ± 8.90
Heart rate (bpm)	79.20 ± 10.2 NS A/NS B	81.40 ± 9.70 NS C	80.70 ± 9.90
Aspartate transaminase (AST) (IU/L) (n.v. 8–40)	22.10 ± 3.1 ** A/** B	24.00 ± 2.90 ** C	22.00 ± 2.80
Alanine transaminase (ALT) (IU/L) (n.v. 8–40)	20.10 ± 2.20 ** A/** B	19.00 ± 2.80 * C	18.00 ± 2.90
Gamma-glutamyl transferase (γGT) (IU/l) (n.v. 5–30)	25.80 ± 4.10 ** A/** B	27.00 ± 3.40 * C	28.00 ± 3.40
Alkaline phosphatase (IU/L) (n.v. 35–100)	37.80 ± 4.10 ** A/** B	51.00 ± 2.80 ** C	54.00 ± 3.60
Total bilirubin (mg/dL) (n.v. 0.2–1.2)	0.90 ± 0.28 * A/* B	1.06 ± 0.40 NS C	1.07 ± 0.60
Fasting glucose (mg/dL) (n.v. 74–106)	80.10 ± 7.90 ** A/** B	88.00 ± 7.50 * C	86.00 ± 7.60
Serum urea (mg/dL) (n.v.6–40)	34.20 ± 5.00 ** A/** B	36.00 ± 5.10 NS C	37.00 ± 5.30
Serum creatinine (mg/dL) (n.v. 0.7–1.3)	0.78 ± 0.40 NS A/** B	0.70 ± 0.40 ** C	0.60 ± 0.30
CA 19-9 (IU/mL) (n.v. < 39)	40.1 ± 21.80 ** A/** B	69.20 ± 39.20 NS C	68.40 ± 30.30

**Table 3:** Analysis of CA 19-9 serum positivity (n.v. < 39).

	BCC			SCC			Dermatitis		
		CI 95%	CI 99%		CI 95%	CI 99%		CI 95%	CI 99%
Sensitivity (SE)	0.688	(0.597–0.766)	(0.568–0.787)	0.586	(0.514–0.655)	(0.492–0.675)	0.667	(0.612–0.718)	(0.594–0.732)
Specificity (SP)	0.725	(0.636–0.800)	(0.608–0.819)	0.806	(0.742–0.857)	(0.721–0.870)	0.914	(0.876–0.941)	(0.863–0.948)
Positive Predictive Value (PPV)	0.468	(0.378–0.560)	(0.353–0.587)	0.557	(0.485–0.627)	(0.463–0.648)	0.129	(0.095–0.172)	(0.087–0.187)
Negative Predictive Value (NPV)	0.868	(0.793–0.920)	(0.766–0.931)	0.824	(0.761–0.873)	(0.741–0.885)	0.993	(0.974–0.999)	(0.965–0.999)
Positive Likelihood Ratio (LR+)	2.503	(1.919–3.264)	(1.765–3.548)	3.018	(2.395–3.803)	(2.227–4.089)	7.753	(4.399–13.666)	(3.681–16.331)
Negative Likelihood Ratio (LR−)	0.431	(0.330–0.562)	(0.304–0.611)	0.514	(0.408–0.647)	(0.379–0.696)	0.365	(0.207–0.643)	(0.173–0.768)
Prevalence	0.26	(0.187–0.348)	(0.169–0.377)	0.294	(0.233–0.364)	(0.216–0.386)	0.019	(0.008–0.042)	(0.006–0.052)
Odds pre-test−	0.352	-	-	0.417	-	-	0.019	-	-
Odds post-test+	0.88	-	-	1.259	-	-	0.148	-	-
Odds post-test−	0.152	-	-	0.214	-	-	0.007	-	-
P pre-test	0.26	(0.115–0.419)	(0.077–0.467)	0.294	(0.180–0.416)	(0.148–0.453)	0.019	(−0.085–0.133)	(−0.113–0.170)
P post-test+	0.468	(0.339–0.598)	(0.303–0.636)	0.557	(0.462–0.650)	(0.433–0.677)	0.129	(0.031–0.236)	(0.003–0.270)
P post-test−	0.132	(−0.022–0.307)	(−0.061–0.361)	0.176	(0.055–0.310)	(0.022–0.351)	0.007	(−0.097–0.122)	(−0.126–0.159)

**Table 4:** Occult malignancies per site in NMSC and dermatitis with high levels of CA 19-9.

		BCC		SCC		Dermatitis
	N	%	N	%	N	%
Pancreas	14	23.33	16	16.67	3	16.67
Breast	10	16.67	14	14.58	5	27.78
Prostate	11	18.33	18	18.75	1	5.56
Bladder	4	6.67	10	10.42	2	11.11
Uterus	4	6.67	6	6.25	2	11.11
Lung	10	16.67	14	14.58	2	11.11
Gallbladder	1	1.67	3	3.13	1	5.56
Stomach	1	1.67	4	4.17	1	5.56
Oesophagus	1	1.67	1	1.04	0	0.00
Colon	2	3.33	4	4.17	1	5.56
Rectum	2	3.33	4	4.17	0	0.00
HCC *	0	0.00	2	2.08	0	0.00
Total cases		60		96		18

* HCC: Hepatocellular Carcinoma

## Data Availability

The dataset supporting the conclusions of this article is included within the tables.
